# Attention demanding tasks during treadmill walking reduce step width variability in young adults

**DOI:** 10.1186/1743-0003-2-25

**Published:** 2005-08-08

**Authors:** Mark D Grabiner, Karen L Troy

**Affiliations:** 1 Department of Movement Sciences, University of Illinois at Chicago, USA

## Abstract

**Background:**

The variability of step time and step width is associated with falls by older adults. Further, step time is significantly influenced when performing attention demanding tasks while walking. Without exception, step time variability has been reported to increase in normal and pathologically aging older adults. Because of the role of step width in managing frontal plane dynamic stability, documenting the influence of attention-demanding tasks on step width variability may provide insight to events that can disturb dynamic stability during locomotion and increase fall risk. Preliminary evidence suggests performance of an attention demanding task significantly decreases step width variability of young adults walking on a treadmill. The purpose of the present study was to confirm or refute this finding by characterizing the extent and direction of the effects of a widely used attention demanding task (Stroop test) on the step width variability of young adults walking on a motorized treadmill.

**Methods:**

Fifteen healthy young adults walked on a motorized treadmill at a self-selected velocity for 10 minutes under two conditions; without performing an attention demanding task and while performing the Stroop test. Step width of continuous and consecutive steps during the collection was derived from the data recorded using a motion capture system. Step width variability was computed as the standard deviation of all recorded steps.

**Results:**

Step width decreased four percent during performance of the Stroop test but the effect was not significant (p = 0.10). In contrast, the 16 percent decrease in step width variability during the Stroop test condition was significant (p = 0.029).

**Conclusion:**

The results support those of our previous work in which a different attention demanding task also decreased step width variability of young subjects while walking on a treadmill. The decreased step width variability observed while performing an attention demanding task during treadmill walking may reflect a voluntary gait adaptation toward a more conservative gait pattern emphasizing frontal plane control of the trunk. Extension of the experimental paradigm to older adults and mechanistic approaches to link step width variability to dynamic stability, and falls, in a cause-effect manner are necessary.

## Introduction

Dynamic stability during locomotion can be negatively affected by concomitant information processing and the effect appears to increase with age [1]. These effects have generally been studied using a dual-task paradigm that introduces performance of an attention demanding secondary task during performance of the primary task, locomotion. The basis of the dual-task paradigm is the assumption that humans possess limited information processing capacity. When simultaneously performing the primary and secondary tasks, each of which require some level of attention, a negative influence on the performance of either task is reflective of task interference [1]. The interference may indicate structural interference or capacity interference. The former is associated with tasks that share common input and output resources whereas the latter is associated with the total information processing capacity having been exceeded.

The use of dual-task paradigms to investigate locomotion is, in part, based on the frequency with which locomotion, generally considered a highly automated motor task, is performed concurrently with cognitive tasks. The changes in reaction time and gait-related variables [e.g., [2-5]] reported for older adults during dual-task paradigms have been associated with increased fall-risk. For example, performing a verbal reaction time task during an obstacle avoidance task significantly increased the risk of obstacle contact by young adults [6]. In another study, a verbal reaction time task increased the risk of obstacle contact by both younger and older adults although the increase was larger in the older adults [7]. These results broadly suggest that performing cognitive tasks during locomotion may increase the risk of tripping. It is notable that secondary task interference on a primary task may be eliminated by practice [8] and, not surprisingly, priority can be directed at the primary task at the expense of decreasing performance on the secondary task [9].

Aging is associated with an increasingly conservative walking pattern marked by changes in basic step kinematics. The changes, which include decreased step length, increased step width and increased double support time, may reduce fall-risk. For example, longer double support time translates to a longer period of time during which the vertical projection of the total body center of mass is within the base of support. A larger step width essentially extends the lateral margins of the base of support and perhaps improves laterally directed control of whole body center of mass position and velocity. The variability of step kinematics has been strongly linked with falls by older adults. In particular, cross-sectional and prospective studies have consistently linked increased step time variability to falls by normally aging [10-12] and pathologically aging older adults [13,14]. Although older adults without a history of falls appear to have increased step width and step width variability compared to young adults [15], a prospective study reported that increased step width and decreased step width variability discriminated older adults who fell from those who did not fall [16]. If viewed as reflecting the presence of noise in a physiological system, variability is deleterious. In this view the decreased step width variability of the older adults who fell is counterintuitive. However, if viewed as arising from multiple interacting control systems, variability has been suggested as reflecting a desirable trait of an adaptive system [17]. From this viewpoint, the diminished step width variability of the older adults who fell is a consistent observation. The biomechanical and physiological significance of altered step width variability (and step time variability), however, has not yet been defined.

The apparent relationship between increased fall-risk when performing an attention demanding task while walking, and the relationship between step kinematic variability and fall risk raises the question of whether attention demanding tasks influence step kinematic variability. There is evidence that the answer is affirmative For example, the step time variability of patients with Parkinson's Disease and Alzheimer's disease, which is significantly larger than that of healthy controls, demonstrates additional and significant increases when walking and performing an attention demanding task [13,14]. Regarding step width variability, in one study performance of an attention demanding task (walking with a cup of water placed in a saucer) did not influence step width variability of either young or older adults [18]. However, methodologic issues may have influenced that result. These issues, specifically related to the technology used to measure step width and number of consecutive steps used to compute step width variability, were subsequently resolved using different instrumentation [19]. Using this technology an attention demanding task (maintaining the beam of an activated laser pointer within a target area) was found to significantly decrease step width variability in young adults [20]. This was a surprising finding in light of the body of literature indicating that increased step time variability is associated with both normal and pathological aging.

Our current interest in step width variability is driven by its role in maintaining laterally directed dynamic stability during gait and its potential utility as a clinical index of laterally directed dynamic stability. Our long-term goal is to determine if subtle changes in step width variability can alter the sensitivity of laterally directed dynamic stability to unexpected postural disturbances and thereby increase fall risk in older adults. The purpose of the present study was to confirm or refute the findings of Walters et al. [20] by characterizing the extent and direction of the effects of an attention demanding task (Stroop test) on the step width variability of young adults during treadmill walking.

## Methods

Fifteen young healthy individuals (8 males and 7 females, age: 24.5 ± 3.4 years, height: 1.66 ± 0.12 m, and mass: 68.5 ± 8.0 kg) volunteered to participate in the study. The protocol was reviewed and approved institutionally and all subjects provided written informed consent prior to participation in the study.

The experiment consisted of three protocols, the order of which was randomly assigned and performed in a single laboratory session. In one protocol, subjects walked on a motorized treadmill at a self-selected speed for 10 minutes. This served as the control walking condition. During the second 10 minute protocol, the subjects walked on the treadmill at the same self-selected speed while performing an attention demanding task [[21], described in the next section]. The third protocol, conducted with the subjects in an upright standing position, provided a baseline measure of performance of the attention demanding task.

During the control walking condition, subjects were asked to walk while looking straight ahead at a wall that was approximately five meters away. The attention demanding task was the Stroop test. During the Stroop test images consisting of the name of one of four colors, printed in text of a different color, were projected onto the wall. The height of the letters, when projected on the wall was 15 cm. The images changed at a frequency of one Hz. The subjects were instructed to verbally identify the color of the text and to ignore the word itself. Incorrect answers were recorded by an investigator. The metric of Stroop test performance was the percentage of wrong answers.

Step width was quantified using motion analysis. The longitudinal axes of the right and left feet were marked using passively reflecting markers placed on the shoe over the heel and the over the third metatarsal. The height of the heel marker was matched to the height of the metatarsal marker. The motion of the reflecting markers was recorded using an eight-camera motion analysis system (Motion Analysis, Santa Rosa, CA, USA) operating at 60 Hz.

For each pair of sequential left-right foot placements, step width was calculated using the midpoints of the foot segments using a custom MATLAB algorithm [22]. Global coordinates were aligned so that the direction of the walk was along the Y-axis and the step width was measured in the X direction. Stance phase was characterized by the period during which the vertical position of the heel markers was approximately zero. Step width was computed as the distance, in the X direction, between the positions of the midpoint of the feet during two sequential stance phases. The midpoint of each foot was calculated as the midpoint of the segment marked by the reflective markers placed over the heel and metatarsal. Step width variability was calculated as the standard deviation of step width from all of the collected steps [19].

The effect of performing the Stroop test on step width and step width variability was determined by comparing the values to those of the control walking condition using paired t-tests. Paired t-tests were used to compare the error rate on the Stroop test during the upright standing condition to that during the control walking condition. A Pearson correlation was calculated to describe the relationship between the error rates on the Stroop test during the control walking and standing conditions. All analyses were performed using SPSS (Version 12.0).

## Results

**Figure 1:**
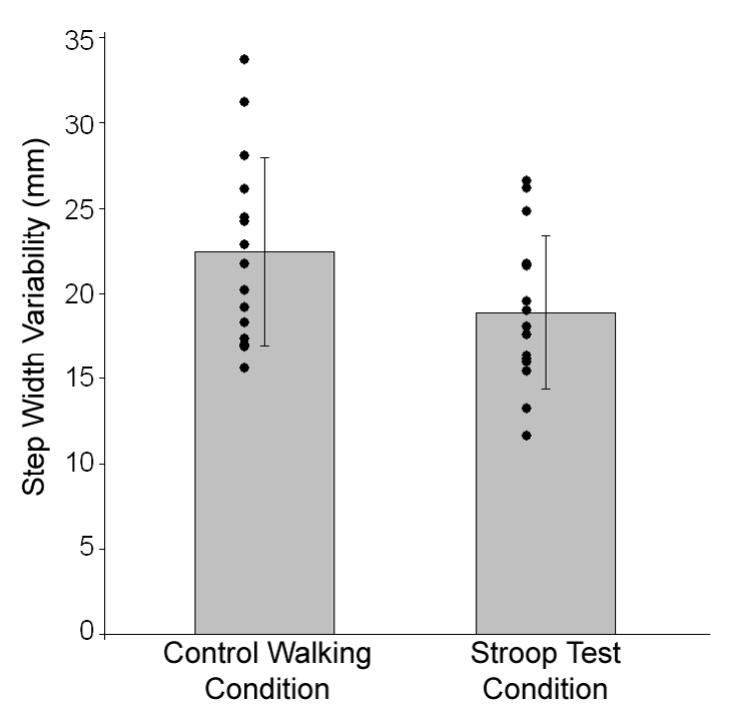
Composite means and standard deviations of step width variability for the control walking condition and the Stroop test condition are illustrated with individual subject data point pairs (n = 15).

Performance of the Stroop test while walking had a significant influence on step width variability. Compared to the control walking condition, step width variability decreased 16 percent while performing the Stroop test (p = 0.029). The step width variability measured during the control condition and the Stroop test condition was 22.4 ± 5.5 mm and 18.9 ± 4.5 mm, respectively (Figure 1).

In contrast to step width variability, performing the Stroop test did not affect step width. Compared to the control walking condition, step width decreased four percent while performing the Stroop test (p = 0.10). The step width measured during the control condition and the Stroop test condition was 152.0 ± 28.3 mm and 146.1 ± 27.1 mm, respectively.

Compared to the standing condition, walking on the treadmill appeared to diminish performance on the Stroop test. During the upright standing condition the error rate was 2.4 ± 3.5 percent whereas during the treadmill test the error rate more than doubled to 5.2 ± 4.7 percent (p = 0.052). Both values were significantly different than zero as indicated by a one-sample t-test (p = 0.02 and 0.001 for the standing and walking conditions, respectively). Notably, there was virtually no relationship between the error rates on the Stroop test during the control walking and standing conditions (r = 0.01, p = 0.73).

## Discussion

The purpose of the present study was to confirm or refute a previous finding of decreased step width variability while walking on a treadmill and performing an attention demanding task [20]. The results confirm those previous results by demonstrating a significant decrease in the step width variability of young adults performing the Stroop test while walking on a treadmill. In the previous study [20], maintaining the beam of a handheld laser pointer within the boundary of a target placed about two meters in front of the subject was associated with a 12.2 percent decrease in step width variability; from 20.5 ± 4.1 mm to 18.0 ± 3.8 mm (p < 0.001). These values bear notable similarity to those observed in the present work.

The findings appear consistent in context with a number of related published studies. Performance of attention demanding tasks increases fall-risk by older adults (2–5). In addition, decreased step width variability distinguished older adults who, in a prospective study, fell from those who did not fall (10). Thus, the present results may implicate performance of attention demanding tasks with changes to a characteristic of gait previously associated with falls by older adults. However, in the absence of any published data related to the influence of performing attention demanding tasks on the step width variability of older adults the implication is indirect.

Despite the consistency of the findings during treadmill walking, however, the present data differ from those of Bauby and Kuo [23] who observed a 53 percent increase in step width variability of young subjects during overground walking with their eyes closed. The amplitude and direction differences between our results and those of Bauby and Kuo may reflect differences in the availability of vision. Humans veer, or deviate from straight line walking, after just a couple of meters without vision [24]. It is possible in the experiment of Bauby and Kuo, during which subjects received verbal stimuli to help them maintain a straight line gait, that the increased step width variability resulted from an interaction between the veering due to the absence of vision and the corrections in response to the verbal stimuli. In the present study, visual information was not absent although the extent to which it was available for guidance may have been reduced due to need to direct vision at the projection of the Stroop test words. Thus, the between-study differences in protocols render meaningful comparison of the results difficult. However, the biomechanical and physiological significance of these disparate findings may have considerable clinical importance. It is possible that directional changes (increase vs. decrease) are contextual and must be considered relative to the specific experimental conditions. In addition, it may be that changes in step width variability can not be considered in isolation from other relevant variables. For example, in the present study and that of Walter et al. [20] there was no effect of the attention demanding task on step width. In contrast, in the study of Bauby and Kuo step width increased by 11 percent. In the work of Maki [16], the older adults who fell demonstrated increased step width (compared to young adults) and decreased step width variability compared to older adults who did not fall.

A question raised by the present results is whether decreased step width variability, an outcome of performing an attention demanding task during treadmill walking, is causally linked to falls in the same manner as is apparent in the results of Maki [16]. From an empirical standpoint, step width variability may represent a manifestation of a mechanism underlying frontal plane control of the trunk. For example, external pelvic stabilization significantly reduced step width variability of young subjects walking on a treadmill by 33 percent [25]. This accompanied a 60 percent decrease in the peak lateral displacement of the center of mass, which implies a reduction in the amplitude of the trunk motion. In other studies, step width variability was significantly reduced while walking with a cane and maintaining contact between a hand and a wall [26] and grasping handles while walking on a motorized treadmill [15]; all of which would be expected to decrease the amplitude of frontal plane trunk motion. If so, then step width variability would be expected to parallel changes in the variability of frontal plane trunk motion. It merits mention that although recent data argue to the contrary [27] pilot data in our laboratory suggest a strong and statistically significant relationship between step width variability and the variability of frontal plane trunk kinematics.

Subtle age-related changes in control of step width may be associated with similarly subtle changes in frontal plane trunk control. The mass of the trunk and its location relative to the base of support during gait underscores the need for active dynamic stabilization in the lateral direction [23]. Disturbances that influence the position, velocity and acceleration of the trunk relative to the base of support, could lead to potentially deleterious biomechanical events. Further investigation of the relationship between step kinematic variability, biomechanics of the trunk, and motor control of the trunk seem warranted. This is particularly relevant for older adults, for whom control of the trunk appears to be decreased. For example, when subjected to a 7.5 degree laterally directed tilt of the platform on which they stood, the trunk of young subjects moved in the direction opposite to that of the tilt within 30 milliseconds [28]. In contrast, the response latency of the older adults was greater than 150 milliseconds and the subsequent trunk motion was in the direction of the impending fall.

If decreased step width variability is associated with decreased frontal plane trunk motion, it may be reasonable to expect that decreased step width variability reflects increased dynamic stability. If so, performance of the attention demanding task in the present study may have caused subjects to adopt a more conservative gait pattern, implying an increase in the voluntary control of gait. This makes sense given the visual resources invested in performing the Stroop test. Reduced availability of visually-derived information of the limbs and treadmill may increase uncertainty about foot placement. Given that a step causing a foot to be placed to some extent off the treadmill belt would be a destabilizing event that subjects could be expected to want to avoid. Indeed, one might speculate that the potential for a considerable destabilizing event might be associated with increased trunk stiffness, a condition that can significantly increase the risk for laterally directed falls [25,29]. Thus, it is proposed that in this manner, decreased step width variability reflects decreased dynamic stability.

The published work related to the influence of an attention demanding task on step width variability is quite limited. However, there is a considerable body of literature related to step time variability as it relates to normal and pathological aging. This literature consistently reports *increased *step time variability in older adults with a history of falls [10], patients with Huntington's disease [30], Parkinson's disease [14,30] and cardiovascular disease [31]. Notably, healthy older adults have been reported to have step time variability that is not different from that of young adults [10] although step width variability of healthy older adults is significantly larger than that of young adults [15]. The functional meaning of the directional differences in the effect of performing an attention demanding task on step time variability (increased variability) and step width variability (decreased variability) have not been resolved at this time. However, the opposite directions in which the changes occur provide an impetus to more fully investigate the relationship between changes in spatial and temporal step kinematic variability as well as the extent to which these variables provide dependent or independent information related to the neuromuscular control gait. Further work that characterizes the mechanisms by which subtle changes in step time variability and step width variability can be causally related to falls by older adults seems warranted.

Two methodological issues, which limit the extent to which results may be generalized, appear to warrant further study. The first relates to the uncertainty of the extent to which step kinematic variability measured during treadmill walking reflects that measured during unrestricted overground walking. Previous work has suggested that with respect to the variability of spatial step kinematics treadmill walking may be an acceptable representation of overground walking [32]. In light of the need to acquire hundreds of continuous steps for the accurate calculation of step kinematic variability [19,23] the methodological solutions to the question, although available, have yet to be applied. From the standpoint of clinical utility, it is not necessary for treadmill walking to perfectly represent overground walking. It may be sufficient for treadmill walking to be a reliable and valid surrogate for overground walking.

The second issue, perhaps the more easily addressed of the two, relates to the present study having been limited to young subjects. Clearly, the danger of falls and fall-related injuries is an issue that is of greatest interest as it relates to older adults. Because the magnitude of the effect of performing attention demanding tasks, and thus fall-risk, increases with age [7] the present results provide the impetus to extend the hypotheses and method to older adults. Our previous experience provides a basis for the expectation that healthy older subjects will demonstrate decreased step width variability under the described experimental conditions.

In conclusion, step width variability of young adults has been shown to be significantly decreased by the concurrent performance of an attention demanding task. This finding is consistent with our previous pilot work and may have important clinical ramifications. Because step width variability reflects frontal plane dynamic stability, disturbances to step width variability could reflect increased fall-risk. If this is so, measures of step width variability could potentially provide the means to clinically track age-related changes to and the effects of interventions on dynamic stability. To that end, mechanistic studies linking step width variability changes to altered dynamic stability, and falls, in a cause-effect manner are necessary.

## Authors' contributions

MDG conceived the study, evaluated the data and results and was responsible for the initial drafting of the manuscript.

KLT wrote/modified software necessary for the analysis and was involved in drafting and revising the manuscript.

Both authors read and approved the final manuscript.
